# Supplementary material for: Multi‐dataset identification of innovative feature genes and molecular mechanisms in keratoconus
Source: J Cell Mol Med. 2024 Sep 19;28(18):e70079. doi: 10.1111/jcmm.70079 (PMC11412914; doi:10.1111/jcmm.70079)
Supplement: Supplementary file 2 — Material S2. [file JCMM-28-e70079-s002.zip › 7.GSEA analysis/3.DBNDD1/KEGG_FOCAL_ADHESION.html]

Details for gene set KEGG\_FOCAL\_ADHESION[GSEA]

|  || Dataset | DBNDD1.group.cls#h\_versus\_l |
| Phenotype | group.cls#h\_versus\_l |
| Upregulated in class | l |
| GeneSet | KEGG\_FOCAL\_ADHESION |
| Enrichment Score (ES) | -0.38531536 |
| Normalized Enrichment Score (NES) | -1.310085 |
| Nominal p-value | 0.14484127 |
| FDR q-value | 0.7523839 |
| FWER p-Value | 0.96 |
Table: GSEA Results Summary

  

Fig 1: Enrichment plot: KEGG\_FOCAL\_ADHESION      
 Profile of the Running ES Score & Positions of GeneSet Members on the Rank Ordered List

  

| SYMBOL | TITLE | RANK IN GENE LIST | RANK METRIC SCORE | RUNNING ES | CORE ENRICHMENT || 1 | PIK3R5 | na | 35 | 0.708 | 0.0307 | No |
| 2 | RAC3 | na | 1824 | 0.312 | -0.0070 | No |
| 3 | COL1A1 | na | 2101 | 0.293 | -0.0019 | No |
| 4 | PDGFRA | na | 3213 | 0.243 | -0.0232 | No |
| 5 | HGF | na | 3455 | 0.233 | -0.0197 | No |
| 6 | ELK1 | na | 3923 | 0.216 | -0.0235 | No |
| 7 | COL1A2 | na | 3946 | 0.215 | -0.0145 | No |
| 8 | COL2A1 | na | 3982 | 0.214 | -0.0059 | No |
| 9 | COL6A3 | na | 4851 | 0.185 | -0.0228 | No |
| 10 | MYL5 | na | 4993 | 0.181 | -0.0187 | No |
| 11 | TNR | na | 5412 | 0.167 | -0.0233 | No |
| 12 | LAMB4 | na | 5688 | 0.159 | -0.0242 | No |
| 13 | PRKCG | na | 6020 | 0.150 | -0.0270 | No |
| 14 | SHC3 | na | 6808 | 0.129 | -0.0440 | No |
| 15 | PIK3R1 | na | 6912 | 0.126 | -0.0414 | No |
| 16 | COL3A1 | na | 7100 | 0.122 | -0.0413 | No |
| 17 | FLNB | na | 7307 | 0.118 | -0.0420 | No |
| 18 | ITGA4 | na | 7459 | 0.114 | -0.0412 | No |
| 19 | BCAR1 | na | 7587 | 0.112 | -0.0399 | No |
| 20 | LAMC3 | na | 7702 | 0.110 | -0.0383 | No |
| 21 | VWF | na | 7777 | 0.108 | -0.0356 | No |
| 22 | IGF1R | na | 8164 | 0.100 | -0.0422 | No |
| 23 | THBS2 | na | 8340 | 0.097 | -0.0429 | No |
| 24 | SOS1 | na | 8586 | 0.093 | -0.0458 | No |
| 25 | SPP1 | na | 8750 | 0.090 | -0.0465 | No |
| 26 | IBSP | na | 9047 | 0.085 | -0.0513 | No |
| 27 | PDGFA | na | 9340 | 0.081 | -0.0561 | No |
| 28 | AKT2 | na | 9388 | 0.080 | -0.0539 | No |
| 29 | COL6A1 | na | 9438 | 0.079 | -0.0518 | No |
| 30 | PARVG | na | 9522 | 0.078 | -0.0507 | No |
| 31 | LAMA2 | na | 9618 | 0.077 | -0.0500 | No |
| 32 | CCND1 | na | 9676 | 0.076 | -0.0482 | No |
| 33 | ITGB7 | na | 9817 | 0.074 | -0.0490 | No |
| 34 | MAPK3 | na | 9865 | 0.073 | -0.0471 | No |
| 35 | PDGFRB | na | 10046 | 0.071 | -0.0491 | No |
| 36 | RASGRF1 | na | 10469 | 0.065 | -0.0584 | No |
| 37 | MAPK10 | na | 10497 | 0.065 | -0.0563 | No |
| 38 | IGF1 | na | 10555 | 0.064 | -0.0551 | No |
| 39 | VAV2 | na | 10635 | 0.063 | -0.0545 | No |
| 40 | PTEN | na | 10959 | 0.059 | -0.0613 | No |
| 41 | COL4A1 | na | 11293 | 0.055 | -0.0685 | No |
| 42 | LAMA5 | na | 11354 | 0.054 | -0.0678 | No |
| 43 | MAPK9 | na | 11537 | 0.052 | -0.0707 | No |
| 44 | PDPK1 | na | 11670 | 0.050 | -0.0723 | No |
| 45 | PIK3R2 | na | 11699 | 0.050 | -0.0709 | No |
| 46 | ROCK1 | na | 11947 | 0.048 | -0.0759 | No |
| 47 | XIAP | na | 12176 | 0.045 | -0.0804 | No |
| 48 | PPP1CA | na | 12524 | 0.042 | -0.0886 | No |
| 49 | THBS4 | na | 12610 | 0.042 | -0.0892 | No |
| 50 | ACTN4 | na | 12890 | 0.039 | -0.0955 | No |
| 51 | ERBB2 | na | 13132 | 0.037 | -0.1008 | No |
| 52 | SOS2 | na | 13580 | 0.033 | -0.1122 | No |
| 53 | PIK3R3 | na | 14144 | 0.029 | -0.1273 | No |
| 54 | PAK4 | na | 14233 | 0.028 | -0.1285 | No |
| 55 | FLT4 | na | 14460 | 0.027 | -0.1339 | No |
| 56 | ACTB | na | 14636 | 0.025 | -0.1378 | No |
| 57 | VTN | na | 14676 | 0.025 | -0.1378 | No |
| 58 | RAC1 | na | 14759 | 0.025 | -0.1391 | No |
| 59 | MYL9 | na | 14830 | 0.024 | -0.1400 | No |
| 60 | FN1 | na | 14969 | 0.023 | -0.1430 | No |
| 61 | PAK6 | na | 14984 | 0.023 | -0.1424 | No |
| 62 | DOCK1 | na | 15109 | 0.022 | -0.1450 | No |
| 63 | ARHGAP35 | na | 15194 | 0.021 | -0.1464 | No |
| 64 | BRAF | na | 15471 | 0.019 | -0.1536 | No |
| 65 | COL5A3 | na | 15822 | 0.017 | -0.1629 | No |
| 66 | FLT1 | na | 15887 | 0.017 | -0.1640 | No |
| 67 | PIP5K1C | na | 16052 | 0.015 | -0.1681 | No |
| 68 | CTNNB1 | na | 16378 | 0.013 | -0.1769 | No |
| 69 | ITGA2B | na | 16436 | 0.013 | -0.1780 | No |
| 70 | VASP | na | 16674 | 0.011 | -0.1843 | No |
| 71 | VAV3 | na | 16809 | 0.011 | -0.1877 | No |
| 72 | RAP1A | na | 16884 | 0.010 | -0.1894 | No |
| 73 | PIK3CA | na | 17073 | 0.009 | -0.1945 | No |
| 74 | MYL12B | na | 17399 | 0.007 | -0.2036 | No |
| 75 | VEGFB | na | 17569 | 0.006 | -0.2082 | No |
| 76 | ACTN2 | na | 17576 | 0.006 | -0.2081 | No |
| 77 | MYL12A | na | 17584 | 0.006 | -0.2080 | No |
| 78 | CCND2 | na | 17929 | 0.003 | -0.2179 | No |
| 79 | PPP1CB | na | 17957 | 0.003 | -0.2185 | No |
| 80 | MYLK2 | na | 17990 | 0.003 | -0.2193 | No |
| 81 | RAP1B | na | 18018 | 0.003 | -0.2200 | No |
| 82 | ACTG1 | na | 18138 | 0.002 | -0.2233 | No |
| 83 | KDR | na | 18209 | 0.001 | -0.2253 | No |
| 84 | TNXB | na | 18316 | 0.001 | -0.2284 | No |
| 85 | PIK3CB | na | 18338 | 0.000 | -0.2289 | No |
| 86 | LAMA4 | na | 18632 | -0.002 | -0.2373 | No |
| 87 | HRAS | na | 18984 | -0.004 | -0.2473 | No |
| 88 | RAF1 | na | 19005 | -0.005 | -0.2477 | No |
| 89 | AKT1 | na | 19066 | -0.005 | -0.2492 | No |
| 90 | RHOA | na | 19096 | -0.005 | -0.2498 | No |
| 91 | EGF | na | 19333 | -0.007 | -0.2563 | No |
| 92 | BIRC2 | na | 19822 | -0.011 | -0.2699 | No |
| 93 | FLNC | na | 20050 | -0.013 | -0.2759 | No |
| 94 | CAPN2 | na | 20301 | -0.016 | -0.2825 | No |
| 95 | TLN2 | na | 20347 | -0.016 | -0.2830 | No |
| 96 | BAD | na | 20453 | -0.017 | -0.2853 | No |
| 97 | ITGB4 | na | 20511 | -0.017 | -0.2862 | No |
| 98 | LAMB2 | na | 20547 | -0.018 | -0.2864 | No |
| 99 | DIAPH1 | na | 20576 | -0.018 | -0.2864 | No |
| 100 | PPP1CC | na | 20793 | -0.020 | -0.2918 | No |
| 101 | RAC2 | na | 20865 | -0.021 | -0.2929 | No |
| 102 | ROCK2 | na | 21182 | -0.024 | -0.3010 | No |
| 103 | ZYX | na | 21218 | -0.024 | -0.3009 | No |
| 104 | VCL | na | 21288 | -0.025 | -0.3018 | No |
| 105 | ITGA9 | na | 21410 | -0.026 | -0.3041 | No |
| 106 | PRKCA | na | 21412 | -0.026 | -0.3030 | No |
| 107 | THBS3 | na | 21883 | -0.031 | -0.3152 | No |
| 108 | ITGB5 | na | 21935 | -0.031 | -0.3153 | No |
| 109 | EGFR | na | 22041 | -0.033 | -0.3169 | No |
| 110 | GRB2 | na | 22294 | -0.035 | -0.3226 | No |
| 111 | CCND3 | na | 22320 | -0.035 | -0.3217 | No |
| 112 | GSK3B | na | 22452 | -0.037 | -0.3239 | No |
| 113 | THBS1 | na | 22482 | -0.037 | -0.3231 | No |
| 114 | MAPK8 | na | 22620 | -0.039 | -0.3253 | No |
| 115 | CDC42 | na | 22626 | -0.039 | -0.3237 | No |
| 116 | MAPK1 | na | 22705 | -0.040 | -0.3242 | No |
| 117 | ILK | na | 22715 | -0.040 | -0.3227 | No |
| 118 | CRK | na | 23147 | -0.045 | -0.3331 | No |
| 119 | ARHGAP5 | na | 23492 | -0.049 | -0.3409 | No |
| 120 | COMP | na | 23752 | -0.053 | -0.3460 | No |
| 121 | PAK3 | na | 23893 | -0.055 | -0.3476 | No |
| 122 | SHC1 | na | 23972 | -0.056 | -0.3473 | No |
| 123 | COL6A2 | na | 23987 | -0.056 | -0.3452 | No |
| 124 | PPP1R12A | na | 24009 | -0.056 | -0.3433 | No |
| 125 | ITGA3 | na | 24015 | -0.056 | -0.3409 | No |
| 126 | JUN | na | 24077 | -0.057 | -0.3401 | No |
| 127 | PTK2 | na | 24353 | -0.061 | -0.3453 | No |
| 128 | PAK2 | na | 24360 | -0.061 | -0.3428 | No |
| 129 | BIRC3 | na | 24545 | -0.064 | -0.3452 | No |
| 130 | COL11A1 | na | 24805 | -0.068 | -0.3497 | No |
| 131 | PIK3CD | na | 24971 | -0.070 | -0.3513 | No |
| 132 | LAMC1 | na | 25472 | -0.078 | -0.3623 | No |
| 133 | MYL2 | na | 25552 | -0.079 | -0.3610 | No |
| 134 | COL11A2 | na | 25749 | -0.083 | -0.3630 | No |
| 135 | ITGAV | na | 25782 | -0.083 | -0.3601 | No |
| 136 | CHAD | na | 25875 | -0.085 | -0.3590 | No |
| 137 | ITGA11 | na | 26521 | -0.098 | -0.3733 | No |
| 138 | ITGA2 | na | 26752 | -0.102 | -0.3753 | No |
| 139 | LAMB1 | na | 26781 | -0.103 | -0.3715 | No |
| 140 | RAPGEF1 | na | 26791 | -0.103 | -0.3671 | No |
| 141 | CRKL | na | 27010 | -0.108 | -0.3686 | No |
| 142 | TNN | na | 27130 | -0.111 | -0.3671 | No |
| 143 | PIK3CG | na | 27455 | -0.118 | -0.3712 | No |
| 144 | RELN | na | 27900 | -0.129 | -0.3782 | No |
| 145 | PAK1 | na | 28053 | -0.132 | -0.3767 | No |
| 146 | MYL7 | na | 28100 | -0.133 | -0.3721 | No |
| 147 | PARVA | na | 28559 | -0.145 | -0.3788 | Yes |
| 148 | MYLK3 | na | 28605 | -0.146 | -0.3735 | Yes |
| 149 | LAMA3 | na | 28652 | -0.148 | -0.3683 | Yes |
| 150 | ITGA6 | na | 28732 | -0.150 | -0.3638 | Yes |
| 151 | MYLK | na | 28743 | -0.150 | -0.3574 | Yes |
| 152 | SRC | na | 28777 | -0.151 | -0.3516 | Yes |
| 153 | VAV1 | na | 28859 | -0.153 | -0.3471 | Yes |
| 154 | COL4A4 | na | 28877 | -0.154 | -0.3407 | Yes |
| 155 | COL4A2 | na | 28922 | -0.155 | -0.3350 | Yes |
| 156 | ITGA7 | na | 29075 | -0.159 | -0.3323 | Yes |
| 157 | ITGB1 | na | 29102 | -0.160 | -0.3258 | Yes |
| 158 | LAMC2 | na | 29103 | -0.160 | -0.3187 | Yes |
| 159 | PXN | na | 29171 | -0.162 | -0.3133 | Yes |
| 160 | LAMB3 | na | 29197 | -0.163 | -0.3068 | Yes |
| 161 | MAP2K1 | na | 29246 | -0.164 | -0.3008 | Yes |
| 162 | ITGB3 | na | 29286 | -0.165 | -0.2945 | Yes |
| 163 | PGF | na | 29370 | -0.168 | -0.2893 | Yes |
| 164 | ITGB6 | na | 29433 | -0.170 | -0.2835 | Yes |
| 165 | MET | na | 29439 | -0.170 | -0.2760 | Yes |
| 166 | VEGFA | na | 30038 | -0.189 | -0.2848 | Yes |
| 167 | PARVB | na | 30086 | -0.191 | -0.2776 | Yes |
| 168 | COL5A2 | na | 30120 | -0.192 | -0.2700 | Yes |
| 169 | FLNA | na | 30291 | -0.197 | -0.2660 | Yes |
| 170 | ACTN1 | na | 30298 | -0.197 | -0.2574 | Yes |
| 171 | ITGB8 | na | 30390 | -0.200 | -0.2510 | Yes |
| 172 | TLN1 | na | 30538 | -0.205 | -0.2461 | Yes |
| 173 | TNC | na | 30547 | -0.205 | -0.2371 | Yes |
| 174 | ITGA1 | na | 31047 | -0.222 | -0.2416 | Yes |
| 175 | FYN | na | 31175 | -0.227 | -0.2350 | Yes |
| 176 | COL6A6 | na | 31239 | -0.230 | -0.2265 | Yes |
| 177 | BCL2 | na | 31549 | -0.242 | -0.2246 | Yes |
| 178 | VEGFC | na | 31739 | -0.251 | -0.2188 | Yes |
| 179 | COL5A1 | na | 31998 | -0.263 | -0.2145 | Yes |
| 180 | ITGA10 | na | 32340 | -0.284 | -0.2116 | Yes |
| 181 | PDGFD | na | 32416 | -0.288 | -0.2009 | Yes |
| 182 | SHC2 | na | 32639 | -0.303 | -0.1937 | Yes |
| 183 | CAV2 | na | 32802 | -0.314 | -0.1843 | Yes |
| 184 | MYL10 | na | 32938 | -0.324 | -0.1737 | Yes |
| 185 | LAMA1 | na | 33178 | -0.343 | -0.1652 | Yes |
| 186 | SHC4 | na | 33253 | -0.349 | -0.1517 | Yes |
| 187 | COL4A6 | na | 33261 | -0.350 | -0.1362 | Yes |
| 188 | CAV1 | na | 33347 | -0.357 | -0.1226 | Yes |
| 189 | AKT3 | na | 33656 | -0.385 | -0.1143 | Yes |
| 190 | ACTN3 | na | 33884 | -0.415 | -0.1022 | Yes |
| 191 | PDGFB | na | 34011 | -0.431 | -0.0865 | Yes |
| 192 | ITGA5 | na | 34082 | -0.440 | -0.0688 | Yes |
| 193 | ITGA8 | na | 34117 | -0.445 | -0.0498 | Yes |
| 194 | PRKCB | na | 34153 | -0.452 | -0.0305 | Yes |
| 195 | PDGFC | na | 34154 | -0.452 | -0.0102 | Yes |
| 196 | CAV3 | na | 34569 | -0.597 | 0.0046 | Yes |
Table: GSEA details [plain text format]

  

Fig 2: KEGG\_FOCAL\_ADHESION      
 Blue-Pink O' Gram in the Space of the Analyzed GeneSet

  

Fig 3: KEGG\_FOCAL\_ADHESION: Random ES distribution      
 Gene set null distribution of ES for **KEGG\_FOCAL\_ADHESION**
